# The transcriptome of the bowhead whale *Balaena mysticetus* reveals adaptations of the longest-lived mammal

**DOI:** 10.18632/aging.100699

**Published:** 2014-11-03

**Authors:** Inge Seim, Siming Ma, Xuming Zhou, Maxim V. Gerashchenko, Sang-Goo Lee, Robert Suydam, John C. George, John W. Bickham, Vadim N. Gladyshev

**Affiliations:** ^1^ Division of Genetics, Department of Medicine, Brigham and Women's Hospital, Harvard Medical School, Boston, MA 02115, USA; ^2^ Department of Wildlife Management, North Slope Borough, Barrow, AK 99723, USA; ^3^ Battelle Memorial Institute, Houston, TX 77079, USA

**Keywords:** bowhead whale, transcriptome, insulin/IGF1-axis, aging, evolution

## Abstract

Mammals vary dramatically in lifespan, by at least two-orders of magnitude, but the molecular basis for this difference remains largely unknown. The bowhead whale *Balaena mysticetus* is the longest-lived mammal known, with an estimated maximal lifespan in excess of two hundred years. It is also one of the two largest animals and the most cold-adapted baleen whale species. Here, we report the first genome-wide gene expression analyses of the bowhead whale, based on the *de novo* assembly of its transcriptome. Bowhead whale or cetacean-specific changes in gene expression were identified in the liver, kidney and heart, and complemented with analyses of positively selected genes. Changes associated with altered insulin signaling and other gene expression patterns could help explain the remarkable longevity of bowhead whales as well as their adaptation to a lipid-rich diet. The data also reveal parallels in candidate longevity adaptations of the bowhead whale, naked mole rat and Brandt's bat. The bowhead whale transcriptome is a valuable resource for the study of this remarkable animal, including the evolution of longevity and its important correlates such as resistance to cancer and other diseases.

## INTRODUCTION

The bowhead whale (*Balaena mysticetus*) is a sentinel baleen whale species of the Arctic [1]. It has a suite of adaptations for life in an intensely cold and ice-bound habitat. These include the thickest blubber of any whale, primarily for lipid storage but also for effective thermoregulation, and a unique bow-shaped head that allows creation of breathing holes by breaking thick sea ice from beneath. Whales belong to the order Cetacea, which includes the suborders Mysticeti (baleen whales) and Odontoceti (toothed whales, including dolphins, porpoises, sperm whales, etc.), and is a subgroup nested within Artiodactyla (even-toed ungulates such as the hippopotamus, camels, pig, cow, etc.) [2, 3].

Cetaceans are generally long-lived species and, similarly to humans, display traits such as delayed sexual maturity, low fecundity and high survival rates. In contrast to their terrestrial relatives within the order Artiodactyla, which feed on a carbohydrate-rich diet, cetaceans subsist on a lipid-rich diet of krill and other small marine animals. The bowhead whale is the longest-lived mammal known, with an estimated maximal lifespan up to 211 years [4, 5]. The bowhead's lifespan far exceeds that of other renowned long-lived species of mammals studied for molecular insights into aging, including the naked mole rat (31 years) (*Heterocephalus glaber*) [6] and Brandt's bat (41 years) (*Myotis brandtii*) [7]. The genomic sequences of only a few cetaceans, including the minke whale (*Balaenoptera acutorostrata*) [8], bottlenose dolphin (*Tursiops truncatus*) [9], killer whale (*Orcinus orca*) [10] and Yangtze River dolphin (baiji; *Lipotes vexillifer*) [11], have been determined. However, limited access to tissues of these animals has precluded detailed analyses of biological functions based on gene expression.

As a first step in identifying such patterns, we present the liver, kidney and heart transcriptomes of the bowhead whale. Comparison of the bowhead whale transcriptome with that of the related minke whale and other mammals enabled us to identify candidate genes for the exceptional longevity of the bowhead whale as well as molecular adaptations to a lipid-rich diet. Analyses of gene sequen-ces and expression patterns also informed on various aspects of the biology and evolution of bowhead whales.

## RESULTS AND DISCUSSION

**Figure 1:**
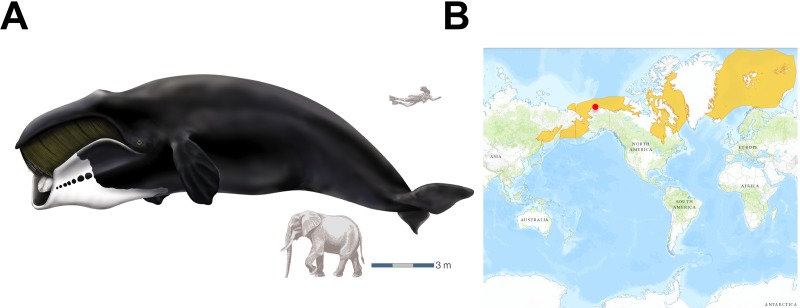
The bowhead whale and overview of its geographic range **(A)** Bowhead whale (*Balaena mysticetus*). Reproduced courtesy of Encyclopaedia Britannica, Inc., copyright 2002. Human and elephant are shown for size comparison. **(B)** Occurrence of *B. mysticetus* in Arctic waters. The site of sample collection (Barrow, Alaska) is shown as a red dot.

**Figure 2:**
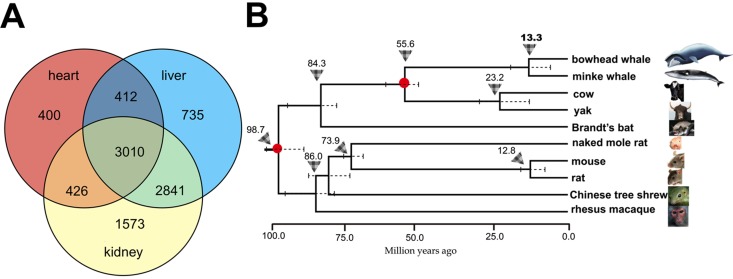
Overview of orthologous genes shared between the bowhead whale and other mammals and the phylogenetic position of the bowhead whale **(A)** Venn diagram showing the number of common and unique orthologs identified in the liver, kidney and heart. **(B)** Estimation of divergence time using phylogenetic analysis of 10 species based on 6,998 orthologous genes from the liver. Triangle arrows and the numbers above denote the most recent common ancestor (MRCA), and the scale units are million years ago. The standard error range for each divergence time is represented by a dashed line. The red solid circles on the branch nodes denote the node as an ‘age constraint’ used in the estimation of the time of divergence.

We sequenced the liver, kidney and heart transcriptomes of bowhead whales by Illumina RNA-seq technology. The tissue samples (Fig. 1 and Supplemental Table 1) were sourced from native Iñupiaq Eskimo subsistence harvests in Barrow, Alaska. Using the *de novo* assembler Trinity [12, 13], approximately 659 million paired-end short reads from the bowhead whale were assembled into a single transcriptome per tissue (Supplemental Table 2). Using the Ensembl mouse gene dataset [14] as reference, we identified 9,395 protein-coding genes with one-to-one orthology between diverse mammalian taxa, including whales, a bat, rodents, a tree shrew and a primate (Fig. 2A).

Phylogenetic analysis of the gene expression dataset revealed that the bowhead and minke whales cluster within one evolutionary branch, with a sister group comprising the cow and yak. The bowhead whale and minke whale diverged approximately 13.3 million years ago (Mya) (Fig. 2B). The estimated molecular divergence time of bowhead and minke whales is slightly less than the estimates from comprehensive morphological and molecular phylogenetic analyses of cetacean phylogeny, examining both extant and fossil lineages in simultaneous analyses [15, 16]. It is thus likely that the common ancestor of the bowhead and minke whales lived approximately 13-27 million years ago.

To reveal candidate genes important in the bowhead's longevity, we compared the bowhead whale transcriptome with that of the minke whale (maximum lifespan of 50 years in the wild [17]), cow, yak, Brandt's bat, Chinese tree shrew, naked mole rat, rhesus macaque, mouse and rat. We also identified unique amino acid changes and rapidly evolving genes in the bowhead whale that may contribute to slow aging in this species.

### Liver

**Figure 3:**
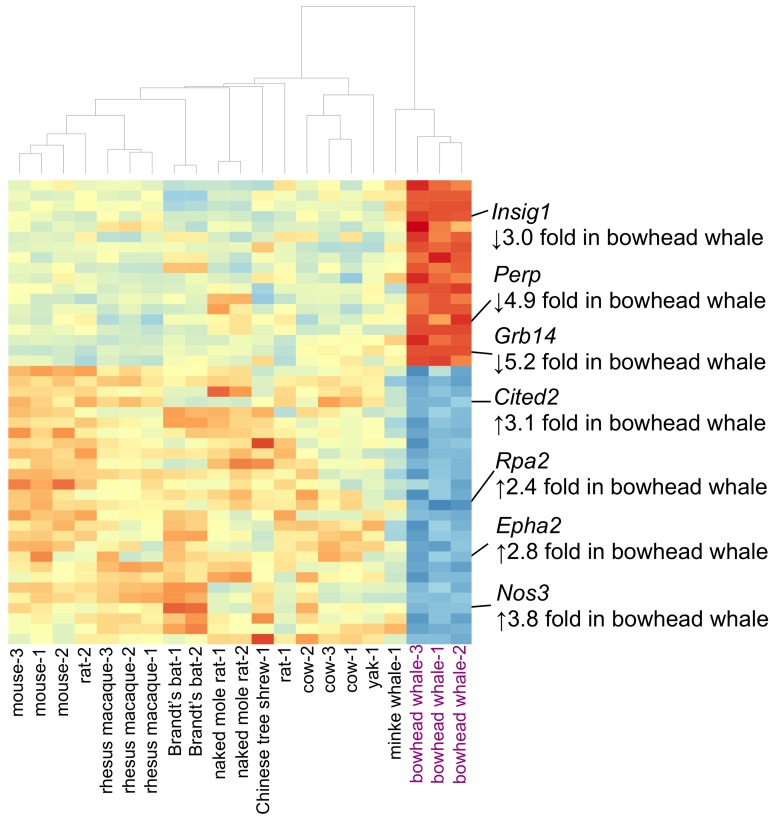
Heat map displaying 45 genes differentially expressed in the bowhead whale liver Genes with ≥2 log2-fold change and with a Benjamini–Hochberg false discovery rate ≤ 0.05) are shown. The color scheme was used wherein the gradient of red indicates decreased expression, and blue increased expression. Selected genes discussed in the text are highlighted. Hierarchical clustering of samples was based on Pearson's correlation of gene expression data.

**Figure 4:**
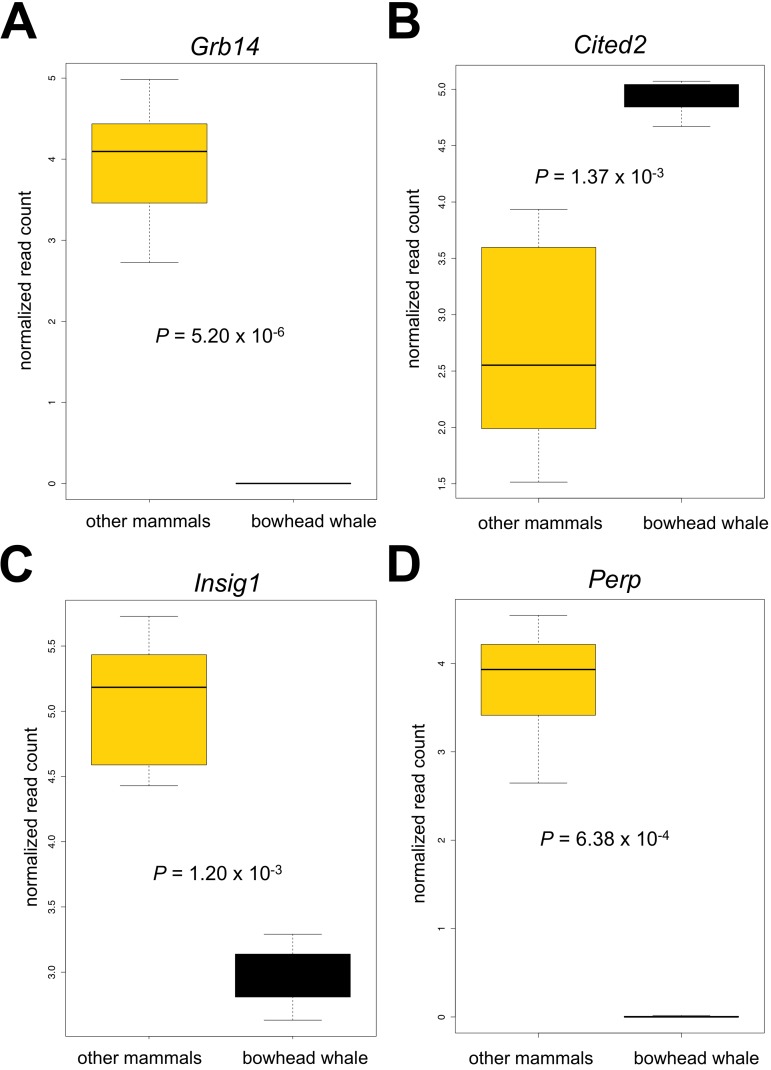
Boxplots of selected genes differentially expressed in the bowhead whale liver **(A)** Growth factor receptor bound protein 14 (*Grb14*). **(B)** Insulin induced gene 1 (*Insig1*). **(C)** Cbp/p300-interacting transactivator (*Cited2*). **(D)** TP53 Apoptosis Effector (*Perp*). Genes were subjected to limma analysis at 5% FDR and 2.0 fold-change cut-off. Normalized log2 read counts and P-values adjusted for multiple comparisons (Benjamini and Hochberg) are shown.

The liver performs several functions vital to whole-body homeostasis. The integrity of liver function is thus important to longevity, and this organ has gained considerable attention in aging research. A total of 45 genes were differentially expressed in the bowhead whale liver compared to other mammals (Fig. 3, and Tables 1 and 2). In particular, the bowhead whale showed greatly reduced expression of growth factor receptor-bound protein 14 (*Grb14*) (Fig. 4A). *Grb14* is normally highly expressed in the liver and kidney of rodents and humans [18]. In contrast to the liver, *Grb14* showed moderate expression in the kidney of the bowhead whale (data not shown). GRB14 binds the receptors for insulin (IR) and IGF1 (IGF1R) to modulate downstream signaling [19-22]. Insulin itself decreases endogenous glucose production (gluconeogenesis) and promotes lipogenesis. Knockout of *Grb14* in mouse reduces the amount of circulating insulin and enhances insulin responsiveness in the liver [21]. Similar effects were observed in primary murine hepatocytes, where RNAi knockdown of *Grb14* augmented insulin-activated signaling and associated decreased expression of genes associated with gluconeogenesis [23]. During fasting and calorie restriction hepatic gluconeogenesis is required for tissues that lack enzymes required to metabolize free fatty acids (e.g. the brain) [24]. Elevated expression of *Cited2* was observed in the bowhead whale (Fig. 4B).

**Table 1:** Genes differentially expressed in the liver of bowhead whales compared to other mammals For each gene, gene counts were normalized across all replicates. We used an absolute value of log2 Ratio≥2, a Benjamini-Hochberg corrected P-value≤ 0.05, and a B-value of at least 2.945 (representing a 95% probability that a gene is differentially expressed) as the threshold to judge the significance of gene expression difference between the bowhead whale and other mammals. A negative fold change denotes a higher gene expression compared to the other mammals examined, and vice versa.

Gene symbol (mouse)	Ensembl transcript ID	Name	logFC	P-value	BH-adj. P-value	B-value
*Adamts13*	ENSMUST00000102891	a disintegrin-like and metallopeptidase (reprolysin type) with thrombospondin type 1 motif	−3.84	5.55E-006	8.95E-004	4.06
*Arap3*	ENSMUST00000042944	ArfGAP with RhoGAP domain	−2.40	9.01E-007	1.96E-004	5.81
*Atp6v1b2*	ENSMUST00000006435	ATPase	2.94	1.42E-008	1.34E-005	9.66
*BC027231*	ENSMUST00000048788	cDNA sequence BC027231	−3.12	6.29E-007	1.61E-004	6.15
*Bgn*	ENSMUST00000033741	biglycan	−2.45	8.00E-006	1.13E-003	3.70
*Card10*	ENSMUST00000164826	caspase recruitment domain family	−3.61	2.61E-007	8.67E-005	7.00
*Cited2*	ENSMUST00000038107	Cbp/p300-interacting transactivator	−3.08	1.14E-005	1.37E-003	3.37
*Cxx1b*	ENSMUST00000088778	CAAX box 1B	−3.87	3.26E-007	9.67E-005	6.78
*Cyp2r1*	ENSMUST00000032908	cytochrome P450	3.42	5.80E-007	1.56E-004	6.21
*Dtd1*	ENSMUST00000028917	D-tyrosyl-tRNA deacylase 1	−2.16	6.96E-007	1.64E-004	6.06
*Epg5*	ENSMUST00000044622	ectopic P-granules autophagy protein 5 homolog (C. elegans)	−4.10	2.70E-006	5.26E-004	4.76
*Epha2*	ENSMUST00000006614	Eph receptor A2	−2.76	5.78E-006	8.98E-004	4.03
*Fam129b*	ENSMUST00000028135	family with sequence similarity 129	−2.97	3.10E-007	9.67E-005	6.83
*Fam92a*	ENSMUST00000108285	family with sequence similarity 92	2.96	6.59E-007	1.62E-004	6.09
*Fbln5*	ENSMUST00000021603	fibulin 5	−4.57	5.76E-008	4.65E-005	8.43
*Grb14*	ENSMUST00000028252	growth factor receptor bound protein 14	5.21	4.61E-009	5.20E-006	10.73
*Ift122*	ENSMUST00000112923	intraflagellar transport 122	−3.02	6.41E-006	9.52E-004	3.93
*Igj*	ENSMUST00000087033	immunoglobulin joining chain	−3.30	9.15E-006	1.20E-003	3.59
*Insig1*	ENSMUST00000059155	insulin induced gene 1	3.04	8.94E-006	1.20E-003	3.63
*Ints2*	ENSMUST00000018212	integrator complex subunit 2	−2.85	5.89E-006	8.98E-004	4.01
*Iqsec1*	ENSMUST00000189881	IQ motif and Sec7 domain 1	5.16	2.00E-007	7.52E-005	7.22
*Lgals3*	ENSMUST00000142734	lectin	−3.58	1.15E-007	6.48E-005	7.78
*Lrrfip2*	ENSMUST00000035078	leucine rich repeat (in FLII) interacting protein 2	2.61	2.52E-007	8.67E-005	6.99
*Masp2*	ENSMUST00000052060	mannan-binding lectin serine peptidase 2	3.46	2.63E-009	4.94E-006	11.32
*Med28*	ENSMUST00000156481	mediator complex subunit 28	3.16	1.93E-006	3.89E-004	5.08
*Nemf*	ENSMUST00000021368	nuclear export mediator factor	3.48	3.17E-006	5.77E-004	4.61
*Ngef*	ENSMUST00000068681	neuronal guanine nucleotide exchange factor	5.16	1.26E-007	6.48E-005	7.65
*Nos3*	ENSMUST00000030834	nitric oxide synthase 3	−3.58	7.94E-006	1.13E-003	3.72
*Orc5*	ENSMUST00000030872	origin recognition complex	2.69	1.92E-006	3.89E-004	5.09
*Pdgfrb*	ENSMUST00000115274	platelet derived growth factor receptor	−4.44	3.62E-006	6.38E-004	4.48
*Perp*	ENSMUST00000019998	PERP	4.91	6.10E-010	3.44E-006	12.57
*Pglyrp2*	ENSMUST00000170392	peptidoglycan recognition protein 2	6.23	1.25E-009	3.52E-006	11.93
*Rasip1*	ENSMUST00000057927	Ras interacting protein 1	−4.69	9.75E-008	6.11E-005	7.94
*Rnase4*	ENSMUST00000022428	ribonuclease	3.21	3.60E-007	1.01E-004	6.69
*Rpa2*	ENSMUST00000102561	replication protein A2	−2.42	2.87E-006	5.41E-004	4.70
*Rpusd4*	ENSMUST00000034543	RNA pseudouridylate synthase domain containing 4	−4.13	1.84E-007	7.52E-005	7.33
*Slc25a38*	ENSMUST00000035106	solute carrier family 25	2.90	8.36E-007	1.89E-004	5.87
*Smad2*	ENSMUST00000168423	SMAD family member 2	2.12	1.58E-005	1.81E-003	3.09
*Ssbp4*	ENSMUST00000049908	single stranded DNA binding protein 4	−2.50	8.39E-006	1.15E-003	3.68
*Tie1*	ENSMUST00000047421	tyrosine kinase with immunoglobulin-like and EGF-like domains 1	−3.79	8.04E-008	5.67E-005	8.12
*Timp2*	ENSMUST00000017610	tissue inhibitor of metalloproteinase 2	−5.09	3.71E-009	5.20E-006	11.02
*Top1*	ENSMUST00000109468	topoisomerase (DNA) I	2.18	1.07E-005	1.35E-003	3.45
*Vac14*	ENSMUST00000034190	Vac14 homolog (S. cerevisiae)	−2.27	1.87E-007	7.52E-005	7.32
*Wsb1*	ENSMUST00000017821	WD repeat and SOCS box-containing 1	−4.40	1.57E-007	7.40E-005	7.48
*Zfp143*	ENSMUST00000084727	zinc finger protein 143	−2.43	5.19E-006	8.62E-004	4.13

**Table 2:** Enrichment of biological process (BP) Gene Ontology (GO) terms for genes differentially expressed in the bowhead liver compared to other mammals

GO Category	Term	Count	Genes	Fold Enrichment	Fisher's exact P-value
GO:0001501	skeletal system development	4	*Epha2, Insig1, Lgals3, Pdgfrb*	7.3	2.00E-003
GO:0048568	embryonic organ development	3	*Epha2, Cited2, Insig1*	7.4	7.20E-003
GO:0006260	DNA replication	3	*Orc5, Rpa2, Top1*	6.1	1.30E-002
GO:0040013	negative regulation of locomotion	2	*Arap3, Tie1*	22.1	3.50E-003
GO:0030336	negative regulation of cell migration	2	*Arap3, Tie1*	22.1	3.50E-003
GO:0001570	vasculogenesis	2	*Epha2, Cited2*	20.6	4.00E-003
GO:0051271	negative regulation of cell motion	2	*Arap3, Tie1*	19.2	4.60E-003

CITED2 is upregulated during fasting, increases the activity of transcriptional co-activator PGC-1α,and directly induces hepatic gluconeogenesis [25]. Interestingly, a recent study found that elevated PGC-1αactivity retards aging in *Drosophila* (26) and it has been hypothesized to also influence longevity in mammals (27). These data suggest that gluconeogenesis is sustained in the bowhead whale despite reduced expression of *Grb14*. Surprisingly, knockdown of *Grb14* in mouse hepatocytes also potently inhibits maturation of sterol regulatory element binding transcription factor 1 (*Srebf1*, often denoted as SREBP1) and decreases lipogenesis, rendering lipogenic genes unresponsive to insulin [23]. In agreement, we observed decreased expression of insulin-induced gene 1 (*Insig1*, a downstream target of SREBP1 [23, 28-29]) in the bowhead whale liver (Fig. 4C), indicative of reduced activities along the GRB14/SREBP1 axis.

Calorie restriction and modulation of the insulin/IGF1-signaling pathway are key modulators of aging and have potent effects on lipid homeostasis [30]. Lipid metabolism, therefore, plays a central role in lifespan regulation in metazoan eukaryotes. While carbohydrates are a major contributor to metabolism of terrestrial mammals, marine mammals subsist on a high-energy, lipid-rich diet [31]. Notably, the bowhead whale diet consists of zooplankton with very high lipid content [32, 33]. Toothed whales, such as the common bottlenose dolphin, which has a maximum lifespan of ~52 years in captivity [34], can exhibit pathology that parallels type two diabetes (T2D) and metabolic syndrome of humans. This includes a high prevalence of insulin resistance, fatty liver disease, and chronic overproduction of Very-low-density lipoprotein (VLDL) by the liver (dyslipidemia) [35-37]. Our data suggest that reduced *Grb14* expression in the bowhead whale improves energy homeostasis in an animal that depends on an exceptionally lipid-rich diet. This beneficial adaptation may serve to protect the long-lived bowhead whale against chronic dietary diseases.

It has been proposed that the longevity of large mammals, such as the bowhead whale and elephant, stem from a lack of non-human predators, slow development and concomitant tight control of nutrient sensing pathways, in particular the insulin/TOR signaling axis [38]. Small African mole rats and many bats live in protected environments and are long-lived relative to animals of similar size, but may in contrast have evolved genetic changes that allow them to slow down aging after a comparatively rapid development [38]. Sequence and expression changes of genes involved in hepatic metabolism have been reported for Brandt's bat [39] and naked mole rat [40], two terrestrial species in our dataset that are long-lived compared to related species. Interestingly, in agreement with the previous studies that compared a smaller set of species, we found that *Foxo1* (forkhead box O1) and *Fto* (fat mass and obesity associated) were differentially expressed in liver of the Brandt's bat and naked mole rat, respectively (Supplemental Tables 3-6). The transcription factor FOXO1, like PGC-1α, is a classical mediator of gluconeogenesis in species ranging from worms to mammals, and *Foxo1* and its upstream regulator *Creb1* [24] are expressed at a higher level in the Brandt's bat liver (Supplemental Table 3). Elevated hepatic *Foxo1* mRNA expression in the Brandt's bat reconciles with increased longevity in calorie restricted and growth hormone receptor knockout mice [41], the worm *C. elegans* [42] and the fruit fly *Drosophila melanogaster* [43]. This result strongly suggests that elevated hepatic *Foxo1* expression contributes to the longevity of the Brandt's bat. The naked mole rat has a highly divergent insulin peptide that may be compensated by autocrine/paracrine IGF2 in the adult liver and displays physiological changes consistent with an altered insulin/IGF1 axis [40, 44]. In the naked mole rat, fat mass and obesity associated mRNA (encoded by *Fto*) is expressed at a lower level compared to the other species examined (Supplemental Table 5). *Fto* regulates glucose metabolism in the liver and its reduced expression protects against obesity and metabolic syndrome, and associated pathology such as insulin resistance [45-47]. Taken together, we propose that fundamental longevity-promoting mechanisms in the long-lived bowhead whale, Brandt's bat and naked mole rat stem from overlapping but distinct expression and sequence changes in metabolism genes.

Living under water also entails a unique set of challenges. Compared with the other mammals, the bowhead liver had lower expression of the apoptosis-associated p53 target gene *Perp* (TP53 Apoptosis Effector) (Fig. 4D). Under hypoxic stress, p53 is activated to trigger cellular apoptosis as a protective response, but this may not be appropriate for mammals with only intermittent assess to oxygen. It has been shown in mouse kidney that *Perp* knockdown protected cells from hypoxia-induced apoptosis [48]. Low levels of *Perp* may therefore increase stress tolerance in the bowhead whale liver. In addition, we observed increased expression of genes that enhance vasculature maintenance (EPH receptor A2 (*Epha2*) [49]), regulate vascular tone and proliferation (endothelial nitric oxide synthase (eNOS) (*Nos3*) [50, 51]) and promote DNA repair (replication protein A2 (*Rpa2*) [52, 53]) (Fig. 3, Tables 1 and 2), which may also represent adaptations to limited oxygen availability and water pressure in the aquatic environment.

### Cardiovascular system

Other extraordinarily long-lived species, such as the naked mole rat, possess biological adaptations endowing resilience to aging-related diseases, including cardiovascular disease and cancer [54, 55]. The availability of a single heart sample precluded differential gene expression analysis of the bowhead whale alone. Using transcriptome data from a bowhead whale heart and a minke whale heart [8], we compared these marine mammals to the terrestrial ones and identified four differentially expressed genes in whales (Table 3). Of these, argininosuccinate lyase (*Asl*) is particularly interesting. The expression of *Asl*, which is essential for NO production in the heart [56], was 4.4-fold higher in the whales (Table 3). Reduction of nitrite to nitric oxide (NO) provides cytoprotection of tissues during hypoxic events [57] and serves to preserve cardiac function in the diving and hibernating red-eared slider turtle (*Trachemys scripta elegans*) [58]. Cardiovascular regulation is critical during diving in all marine mammal species, and evidence suggests that hypoxia-sensitive tissues such as the brain and heart have adapted to low oxygen conditions while diving [59]. We speculate that elevated expression of *Asl* improves cardiac metabolism and function of diving cetaceans.

**Table 3:** Genes differentially expressed in the bowhead and minke whale heart compared to other mammals For each gene, gene counts were normalized across all replicates. We used an absolute value of log2 Ratio≥2, a Benjamini-Hochberg corrected P-value≤ 0.05, and a B-value of at least 2.945 (representing a 95% probability that a gene is differentially expressed) as the threshold to judge the significance of gene expression difference between whales and other mammals. A negative fold change denotes a higher gene expression compared to the other mammals examined, and vice versa.

Gene symbol (mouse)	Ensembl transcript ID	Name	logFC	P-value	BH-adj. P-value	B-value
Asl	ENSMUST00000161094	argininosuccinate lyase	−4.40	1.43E-005	1.50E-002	3.19
Brk1	ENSMUST00000035725	BRICK1, SCAR/WAVE actin-nucleating complex subunit	−2.85	5.69E-006	7.93E-003	3.96
Eif3l	ENSMUST00000040518	eukaryotic translation initiation factor 3, subunit L	−2.46	2.28E-006	4.77E-003	4.95
Htatip2	ENSMUST00000085272	HIV-1 tat interactive protein 2, homolog (human)	−6.00	1.40E-007	5.87E-004	6.99

**Figure 5:**
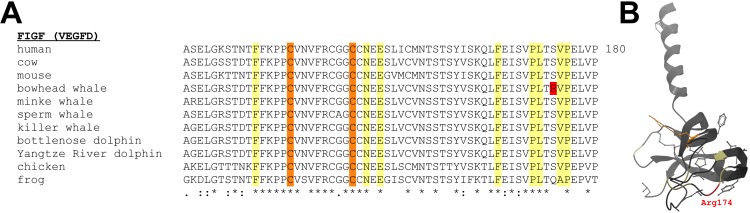
Evolution of vertebrate VEGFD protein sequences **(A)** Alignment of vertebrate VEGFD protein sequences. VEGFD residues at the VEGFR-2 receptor-ligand interface are highlighted in yellow, highly conserved cysteine residues that stabilize VEGFD in orange, and a radical amino acid in the bowhead whale, but not in 67 other species, is shown in red. **(B)** Modeled VEGFD structure. Location of the unique arginine residue in the bowhead whale protein is shown.

Amino-acid residues that are uniquely altered in a lineage can reveal clues to functional adaptations [39, 60]. In addition to differential gene expression patterns, we identified a unique amino acid change with potential relevance to vascular aging in the bowhead whale (Table 4). An examination of 68 vertebrate genomes, including 6 cetaceans (Supplemental Table 7), revealed a unique amino acid change in bowhead whale c-fos induced growth factor (*Figf*, coding for vascular endothelial growth factor D, VEGFD) (Fig. 5). VEGFD plays a role in the maintenance of vascular homeostasis [61, 62]. The radical amino acid change, neutral polar residues replaced by a charged residue (SerThrGln174Arg), in bowhead whale VEGFD flanks a large hydrophobic surface that interacts with the cognate receptor VEGFR-2 [63]. Although this finding must be corroborated with experimental data, we speculate that the arginine substitution may serve to improve the interaction of VEGFD with its cognate receptor, potentially aiding maintenance of vascular health in bowhead whales.

**Table 4:** Proteins with unique amino acids in the bowhead whale compared to 67 vertebrates AA changes correspond to residue locations in the translated human RefSeq mRNA entry (typically the longest RefSeq entry of each gene). Genes shown in bold were checked by BLAST analysis of raw sequence data. Some of the genes in this table, especially those not shown in bold, may be false-positives.

Gene name	Symbol (human)	RefSeq ID	AA change(s)
actin-binding Rho activating protein	*ABRA*	NM_139166	HKR283P
adaptor-related protein complex 3, beta 1 subunit	*AP3B1*	NM_003664	R1086Q
annexin A7	*ANXA7*	NM_001156	Y410C
arachidonate 5-lipoxygenase	*ALOX5*	NM_000698	D204N
calcium homeostasis modulator 2	*CALHM2*	NM_015916	F172C
calsyntenin 2	*CLSTN2*	NM_022131	Q362H
cell division cycle associated 7	*CDCA7*	NM_145810	ED257K
**c-fos induced growth factor (vascular endothelial growth factor D)**	***FIGF***	**NM_004469**	**STQ174R**
ClpB caseinolytic peptidase B homolog (E. coli)	*CLPB*	NM_001258392	HR226W
complement component 5	*C5*	NM_001735	I760K
cytidine and dCMP deaminase domain containing 1	*CDADC1*	NM_030911	YQ367C
derlin 1	*DERL1*	NM_024295	R197G
enhancer of polycomb homolog 1 (Drosophila)	*EPC1*	NM_001272004	G562R
erythrocyte membrane protein band 4.1-like 1	*EPB41L1*	NM_001258329	K478M
fucose-1-phosphate guanylyltransferase	*FPGT*	NM_003838	HK339E
gem (nuclear organelle) associated protein 4	*GEMIN4*	NM_015721	KR721T
gigaxonin	*GAN*	NM_022041	E74V
glutamate receptor, ionotropic, kainate 2	*GRIK2*	NM_175768	Y571C, Q621R
glutamate receptor, metabotropic 1	*GRM1*	NM_001278066	E559D
heat shock protein 90kDa alpha (cytosolic), class B member 1	*HSP90AB1*	NM_001271971	H121T
homeodomain interacting protein kinase 1	*HIPK1*	NM_181358	SP465C
homeodomain interacting protein kinase 3	*HIPK3*	NM_005734	Q698H
ligase III, DNA, ATP-dependent	*LIG3*	NM_013975	D255N
mitogen-activated protein kinase kinase kinase 7	*MAP3K7*	NM_145331	V424D
**MMS19 nucleotide excision repair homolog (S. cerevisiae)**	***MMS19***	**NM_022362**	**SLT202C**
Nance-Horan syndrome (congenital cataracts and dental anomalies)	*NHS*	NM_001291868	R388Q
NEDD4 binding protein 3	*N4BP3*	NM_015111	Q384H
neuronal pentraxin II	*NPTX2*	NM_002523	Q375H
NOP2 nucleolar protein	*NOP2*	NM_001258310	YF516C
NRDE-2, necessary for RNA interference, domain containing	*NRDE2*	NM_017970	H592Q
nuclear receptor coactivator 2	*NCOA2*	NM_006540	F441C
nuclear receptor coactivator 7	*NCOA7*	NM_181782	SAT160D
nucleolar protein 6 (RNA-associated)	*NOL6*	NM_022917	R582L
oncoprotein induced transcript 3	*OIT3*	NM_152635	P353R
oxidative stress induced growth inhibitor family member 2	*OSGIN2*	NM_004337	ED194K
PALM2-AKAP2 readthrough	*PALM2-AKAP2*	NM_147150	ED488G
patched 1	*PTCH1*	NM_000264	S1185C
phosphodiesterase 4D interacting protein	*PDE4DIP*	NM_001002811	SGNTP264R
polymerase (DNA-directed), delta interacting protein 3	*POLDIP3*	NM_032311	Q198R
potassium voltage-gated channel, Shal-related subfamily, member 2	*KCND2*	NM_012281	L457R
protein kinase, cAMP-dependent, regulatory, type I, alpha	*PRKAR1A*	NM_212472	N26D
protocadherin alpha subfamily C, 2	*PCDHAC2*	NM_018899	K884Q
serpin peptidase inhibitor, clade C (antithrombin), member 1	*SERPINC1*	NM_000488	E359D
striatin, calmodulin binding protein	*STRN*	NM_003162	ED279V
transmembrane protein 214	*TMEM214*	NM_017727	R251Q
transmembrane protein 25	*TMEM25*	NM_032780	G162D
tripartite motif containing 29	*TRIM29*	NM_012101	Q250H
tyrosine aminotransferase	*TAT*	NM_000353	Y442H
vacuolar protein sorting 13 homolog B (yeast)	*VPS13B*	NM_015243	G567R

### Kidney

Aging-induced changes in the kidney include reduced repair and/or regeneration of cells, which is concomitant with decreases in glomerular filtration rate and blood flow [64, 65]. Comparison of the bowhead whale kidney transcriptome with those of other mammals revealed that 53 genes were differentially expressed in the bowhead whale kidney (Tables 5 and 6). Among these was a battery of DNA repair associated genes. For example, SprT-like N-terminal domain (*Sprtn*) [66]; non-SMC condensin I complex, subunit D2 (*Ncapd2*) [67]; ligase I, DNA, ATP-dependent (*Lig1*) [68]; and *Rpa2* [52, 53] which also was elevated in the liver, were expressed at higher levels in the bowhead whale kidney (Table 5). The mitochondrial stress inhibitor Junb [69], as well as genes associated with tumor suppression, was also expressed at higher levels in the bowhead whale kidney. These latter genes included E4F transcription factor 1 (*E4fl*) which directly interacts with the tumor suppressors p53, RASSF1A, pRB and p14ARF [70]; ADP-ribosylation factor-like 2 (*Arl2*) [71]; protein kinase C, delta binding protein (*Prkcdbp*; also known as *hSRBC*) [72, 73]; and *Egr1* [74]. We speculate that this series of genes protect against age-related kidney tissue decline in the bowhead whale.

**Table 5:** Genes differentially expressed in the kidney of bowhead whales compared to other mammals For each gene, gene counts were normalized across all replicates. We used an absolute value of log2 Ratio≥2, Benjamini-Hochberg corrected P-value≤ 0.05 a B-value of at least 2.945 (representing a 95% probability that a gene is differentially expressed) as the threshold to judge the significance of gene expression difference between the bowhead whale and other mammals. A negative fold change denotes a higher gene expression compared to the other mammals examined, and vice versa.

Gene symbol (mouse)	Ensembl transcript ID	Name	logFC	P-value	BH-adj. P-value	B-value
*Adamts1*	ENSMUST00000023610	a disintegrin-like and metallopeptidase (reprolysin type) with thrombospondin type 1 motif, 1	−2.08	2.6E-07	1.0E-04	6.98
*Ankrd6*	ENSMUST00000035719	ankyrin repeat domain 6	−3.37	2.5E-06	3.2E-04	4.84
*Arhgef19*	ENSMUST00000006618	Rho guanine nucleotide exchange factor (GEF) 19	−3.41	2.9E-07	1.0E-04	6.89
*Arl2*	ENSMUST00000025893	ADP-ribosylation factor-like 2	−2.36	1.2E-06	2.2E-04	5.52
*Atp5g2*	ENSMUST00000185641	ATP synthase, H+ transporting, mitochondrial F0 complex, subunit C2 (subunit 9)	−2.22	8.6E-07	1.8E-04	5.83
*Cdh3*	ENSMUST00000080797	cadherin 3	−3.41	7.6E-06	6.6E-04	3.73
*Cep170*	ENSMUST00000057037	centrosomal protein 170	−2.71	1.5E-06	2.3E-04	5.36
*Cpsf3l*	ENSMUST00000030901	cleavage and polyadenylation specific factor 3-like	−2.44	3.3E-06	3.9E-04	4.52
*Cxx1b*	ENSMUST00000088778	CAAX box 1B	−2.29	1.6E-06	2.3E-04	5.25
*Dennd4b*	ENSMUST00000098914	DENN/MADD domain containing 4B	−2.68	2.3E-06	3.0E-04	4.92
*E4f1*	ENSMUST00000056032	E4F transcription factor 1	−2.08	1.7E-05	1.1E-03	2.99
*Egr1*	ENSMUST00000064795	early growth response 1	−4.04	2.2E-07	9.4E-05	7.16
*Epb4.1l2*	ENSMUST00000053748	erythrocyte protein band 4.1-like 2	−2.41	2.2E-08	2.0E-05	9.36
*Fancg*	ENSMUST00000030165	Fanconi anemia, complementation group G	−3.22	1.0E-05	7.6E-04	3.51
*Galnt11*	ENSMUST00000045737	UDP-N-acetyl-alpha-D-galactosamine:polypeptide N-acetylgalactosaminyltransferase 11	2.33	7.9E-06	6.6E-04	3.69
*Gja4*	ENSMUST00000053753	gap junction protein, alpha 4	−3.17	7.3E-08	4.8E-05	8.21
*Gltscr2*	ENSMUST00000044158	glioma tumor suppressor candidate region gene 2	−2.60	2.2E-08	2.0E-05	9.40
*Hdac11*	ENSMUST00000041736	histone deacetylase 11	−2.01	8.3E-07	1.8E-04	5.89
*Heatr1*	ENSMUST00000059270	HEAT repeat containing 1	2.60	1.2E-06	2.2E-04	5.47
*Hecw2*	ENSMUST00000120904	HECT, C2 and WW domain containing E3 ubiquitin protein ligase 2	−2.62	7.8E-07	1.8E-04	5.94
*Hoxa5*	ENSMUST00000048794	homeobox A5	−3.44	7.9E-06	6.6E-04	3.73
*Igsf11*	ENSMUST00000023478	immunoglobulin superfamily, member 11	4.09	9.2E-07	1.9E-04	5.72
*Junb*	ENSMUST00000064922	jun B proto-oncogene	−3.44	1.5E-06	2.3E-04	5.29
*Klf9*	ENSMUST00000036884	Kruppel-like factor 9	2.26	9.3E-06	7.2E-04	3.60
*Lig1*	ENSMUST00000177588	ligase I, DNA, ATP-dependent	−2.50	9.3E-06	7.2E-04	3.54
*Lrfn4*	ENSMUST00000113822	leucine rich repeat and fibronectin type III domain containing 4	−2.78	5.0E-07	1.4E-04	6.37
*Marc2*	ENSMUST00000068725	mitochondrial amidoxime reducing component 2	5.01	2.6E-07	1.0E-04	6.90
*Ncapd2*	ENSMUST00000043848	non-SMC condensin I complex, subunit D2	−2.14	1.3E-08	1.5E-05	9.91
*Ncs1*	ENSMUST00000000199	neuronal calcium sensor 1	−2.40	8.7E-07	1.8E-04	5.84
*Nfya*	ENSMUST00000046719	nuclear transcription factor-Y alpha	3.45	2.2E-07	9.4E-05	7.02
*Nipsnap3b*	ENSMUST00000015391	nipsnap homolog 3B (C. elegans)	−2.47	5.0E-06	5.0E-04	4.18
*Nle1*	ENSMUST00000103213	notchless homolog 1 (Drosophila)	−3.32	7.1E-10	2.6E-06	12.57
*Nme6*	ENSMUST00000035053	NME/NM23 nucleoside diphosphate kinase 6	2.34	4.7E-07	1.4E-04	6.30
*Nr4a1*	ENSMUST00000023779	nuclear receptor subfamily 4, group A, member 1	−5.09	1.5E-07	7.7E-05	7.53
*Palld*	ENSMUST00000121785	palladin, cytoskeletal associated protein	4.13	7.0E-09	1.3E-05	10.13
*Pmf1*	ENSMUST00000056370	polyamine-modulated factor 1	−2.39	8.1E-06	6.7E-04	3.72
*Pomk*	ENSMUST00000061850	protein-O-mannose kinase	−2.21	1.0E-06	2.0E-04	5.72
*Por*	ENSMUST00000005651	P450 (cytochrome) oxidoreductase	2.49	4.8E-06	5.0E-04	4.18
*Prelid1*	ENSMUST00000021942	PRELI domain containing 1	2.72	1.4E-07	7.7E-05	7.56
*Prkcdbp*	ENSMUST00000047040	protein kinase C, delta binding protein	−3.03	1.6E-06	2.3E-04	5.25
*Psmg3*	ENSMUST00000031531	proteasome (prosome, macropain) assembly chaperone 3	−2.12	3.8E-06	4.3E-04	4.43
*Rpa2*	ENSMUST00000102561	replication protein A2	−2.61	1.2E-08	1.5E-05	9.95
*Rpl10*	ENSMUST00000008826	ribosomal protein L10	−2.37	7.7E-07	1.8E-04	5.93
*Rps17*	ENSMUST00000080813	ribosomal protein S17	−2.34	1.1E-05	7.9E-04	3.33
*Scg5*	ENSMUST00000024005	secretogranin V	−2.81	6.8E-07	1.7E-04	6.07
*Sema4c*	ENSMUST00000114991	sema domain, immunoglobulin domain (Ig), transmembrane domain (TM) and short cytoplasmic domain, (semaphorin) 4C	−2.55	1.4E-07	7.7E-05	7.58
*Sprtn*	ENSMUST00000034467	SprT-like N-terminal domain	−2.14	1.6E-06	2.3E-04	5.25
*St7*	ENSMUST00000081635	suppression of tumorigenicity 7	2.56	9.5E-06	7.3E-04	3.58
*Syf2*	ENSMUST00000030622	SYF2 homolog, RNA splicing factor (S. cerevisiae)	2.03	1.2E-05	8.6E-04	3.33
*Tle2*	ENSMUST00000146358	transducin-like enhancer of split 2, homolog of Drosophila E(spl)	−2.60	1.6E-06	2.3E-04	5.25
*Traf1*	ENSMUST00000172159	TNF receptor-associated factor 1	−3.11	1.2E-06	2.2E-04	5.56
*Usp9x*	ENSMUST00000089302	ubiquitin specific peptidase 9, X chromosome	5.60	5.1E-11	3.7E-07	14.39
*Zdhhc1*	ENSMUST00000044286	zinc finger, DHHC domain containing 1	−2.16	3.7E-09	8.9E-06	11.09

**Table 6:** Enrichment of biological process (BP) Gene Ontology (GO) terms for genes differentially expressed in the kidney of bowhead whales compared to other mammals

GO Category	Term	Count	Genes	Fold Enrichment	Fisher's exact P-value
GO:0000087	M phase of mitotic cell cycle	8	*Usp9x, E4f1, Pmf1, Ncapd2*	6.1	3.80E-003
GO:0007067	mitosis	8	*Usp9x, E4f1, Pmf1, Ncapd2*	6.1	3.80E-003
GO:0000280	nuclear division	8	*Usp9x, E4f1, Pmf1, Ncapd2*	6.1	3.80E-003
GO:0006350	transcription	20	*Egr1, Klf9, Hoxa5, Nr4a1, Tle2, E4f1, Hdac11, Nfya, Pmf1, Junb*	2.2	1.10E-002
GO:0048285	organelle fission	8	*Usp9x, E4f1, Pmf1, Ncapd2*	5.9	4.40E-003
GO:0000279	M phase	8	*Usp9x, E4f1, Pmf1, Ncapd2*	4.9	8.40E-003
GO:0000278	mitotic cell cycle	8	*Usp9x, E4f1, Pmf1, Ncapd2*	4.8	8.80E-003
GO:0022403	cell cycle phase	8	*Usp9x, E4f1, Pmf1, Ncapd2*	4.2	1.40E-002
GO:0051301	cell division	8	*Usp9x, E4f1, Pmf1, Ncapd2*	4.1	1.60E-002
GO:0001825	blastocyst formation	4	*Nle1, Junb*	24.7	2.80E-003
GO:0045449	regulation of transcription	20	*Egr1, Klf9, Hoxa5, Nr4a1, Tle2, E4f1, Hdac11, Nfya, Pmf1, Junb*	1.8	4.00E-002

Expanding on the theme of improved genome integrity and repair, we found a radical amino acid change SerLeuThr202Cys in the HEAT2 domain of bowhead whale MMS19 nucleotide excision repair homolog (encoded by *Mms19*) (Table 4). This highly conserved protein plays an essential role in genome stability by facilitating iron-sulfur (FeS) cluster insertion into proteins involved in methionine biosynthesis, DNA replication and repair and telomere maintenance [75, 76]. Reactive cysteines in assembly complex proteins, such as MMS19, assist in the transfer of FeS clusters to target proteins [77]. The amino acid change in bowhead whale MMS19 may confer systemic robustness to damage and/or longevity.

### Rapid gene evolution analysis

Identification of positively selected genes can reveal insights into unique adaptations. Of the 9,395 1:1 orthologs from the species shown in Fig. 2B, eight genes showed evidence of positive selection in the bowhead whale lineage (Table 7). Similar to comparisons between human and other primates [78], the small number of positively selected genes in the Arctic bowhead whale may reflect a reduced efficacy of natural selection. Indeed, the bowhead whale has a long generation time (~50 years) and a small population size compared to the common minke whale and the other mammals examined here [79]. Unique amino acid changes were recently identified in cetacean haptoglobin (encoded by *Hp*) [8], a protein that protects against hemoglobin-driven oxidative stress [80]. *Hp* is under positive selection in the bowhead whale (Table 7), suggesting that this gene is rapidly evolving in the long-lived bowhead. Four positively selected genes in the bowhead whale lineage have roles in cancer: mitochondrial tumor suppressor 1 (*Mtus1*) [81]; glycogen synthase kinase 3 alpha (*Gsk3a*) and prune exopolyphosphatase (*Prune*), which act in concert to regulate cell migration [82]; and cytoplasmic FMR1 interacting protein 1 (*Cyfip1*) [83].

**Table 7:** Positively selected genes in the bowhead whale

Gene symbol (mouse)	Ensembl transcript ID	Gene description
*BC026585*	ENSMUST00000046743	quinone oxidoreductase-like protein 2-like
*Cyfip1*	ENSMUST00000032629	cytoplasmic FMR1 interacting protein 1
*Ecm1*	ENSMUST00000117507	extracellular matrix protein 1
*Gsk3a*	ENSMUST00000071739	glycogen synthase kinase 3 alpha
*Gtf2i*	ENSMUST00000059042	general transcription factor II I
*Hp*	ENSMUST00000074898	haptoglobin
*Mtus1*	ENSMUST00000059115	mitochondrial tumor suppressor 1
*Prune*	ENSMUST00000015855	prune exopolyphosphatase

### Concluding comments

Here, we report the transcriptome of the bowhead whale, the longest-lived mammal known. We compared the gene expression of the bowhead whale to shorter-lived mammals, including the minke whale which we estimate to have diverged from a common ancestor approximately 13-27 million years ago (Mya). This is similar to the great apes (gorillas, chimpanzees, orangutans and humans) which share a common ancestor ~13 Mya [84], or human and rhesus macaques which diverged ~25 Mya [85]. Among higher primates, humans are exceptionally long-lived [86]. Similarly, the bowhead whale has increased maximum lifespan compared to related cetaceans. It has been proposed that the difference in longevity between humans and other primates stems from differential expression of a small number of genes [87]. A recent study comparing humans to eight other mammals, including primates, revealed that 93 liver and 253 kidney genes showed evidence of human lineage-specific expression changes [88]. The number of genes differentially expressed in the bowhead whale liver (45 genes) and kidney (53 genes) compared to other mammals is similar, albeit obtained using a different computational method. We speculate that the genes differentially expressed, with unique coding sequence changes and rapidly evolving in the bowhead whale, represent candidate longevity-promoting genes. We particularly stress the findings suggestive of altered insulin signaling and adaptation to a lipid-rich diet. The availability of a single heart tissue sample from the bowhead whale precluded identification of distinct gene expression patterns in the long-lived bowhead whale, but revealed that argininosuccinate lyase (*Asl*) may protect the heart of cetaceans during hypoxic events such as diving. The bowhead whale transcriptome provides valuable resource for further research, including genome annotation and analysis, the evolution of longevity, adaptation to an aquatic environment, conservation efforts and as a reference for closely related species.

## METHODS

### Animals

Kidney (n=4), liver (n=3) and heart (n=1) tissues were obtained from bowhead whales (*Balaena mysticetus*) captured during the 2010 native Iñupiaq Eskimo subsistence harvests in Barrow, AK, USA. See Supplemental Table 1 for details. A species range map, which includes all five stocks of bowhead whales, was obtained from the IUCN Red List of Threatened Species (http://www.iucnredlist.org).

### Transcriptome sequencing

Total RNA was isolated from frozen tissues using a RNAqueous kit (Life Technologies, Carlsland, CA, USA). Short-insert ‘paired-end’ libraries were prepared using the Illumina TruSeq Sample Preparation Kit v2 and Illumina HiSeq 2000 next-generation sequencing was performed following the manufacturer's instructions (Illumina, San Diego, CA, USA). Libraries were sequenced bi-directionally (101 bp in each direction) on the Illumina HiSeq 2000 Genome Analyzer platform.

### *de novo* transcriptome assembly

We obtained and performed RNA-seq on independent biological replicates of bowhead whales (3 liver, 4 kidney and 1 heart samples) (Supplemental Table 1). In addition, publicly available Illumina HiSeq 2000 RNA-seq data were obtained from the following species: the common minke whale *Balaenoptera acutorostrata* (1 liver, 1 kidney, 1 heart) [8], cow *Bos taurus* (3 livers, 3 kidneys, 3 hearts) [89], (domestic) yak *Bos grunniens* (1 liver, 1 heart) [90], Brandt's bat *Myotis brandtii* (2 livers, 2 kidneys – all from summer active bats) [39], naked mole rat *Heterocephalus glaber* (2 livers, 2 kidneys) [91], Chinese tree shrew *Tupaia chinensis* (1 liver, 1 kidney, 1 heart) [92], mouse *Mus musculus* (3 livers, 3 kidneys, 2 hearts) [93], rat *Rattus norvegicus* (2 livers, 3 kidneys, 2 hearts) [89] and rhesus macaque *Macaca mulatta* (3 livers, 3 kidneys, 2 hearts) [89]. Prior to assembly, all reads (in FASTQ format) were filtered for adapter contamination, ambiguous residues (N's) and low quality regions using nesoni v0.123 (http://www.vicbioinformatics.com/software.nesoni.shtml) with default settings. Trimmed read quality was visualized using FastQC (http://www.bioinformatics.bbsrc.ac.uk/projects/fastqc). For each species, trimmed reads from a tissue were pooled and assembled using Trinity v20140717 [12,13] with default parameters. Assembly statistics can be found in Supplemental Table 2.

### Ortholog annotation

To identify orthologous protein-coding transcripts we employed a custom pipeline written in the R statistical computing language [93]. First, we extracted the longest open reading frame for each gene in Mouse Ensembl gene data set GRCm38.p2 (hereafter termed “Reference ORF”) and confirmed they were all in frame, had start and stop codons, and did not have internal stop codons. For genes with multiple alternatively spliced transcripts, the longest transcript was kept. Putative orthologs between the mouse and Trinity assembled transcriptomes (each Trinity transcriptome contains a set of Trinity Transcripts) were identified by a bidirectional best-hit method by discontiguous MegaBLAST. MegaBLAST is bundled in v2.2.29 of the BLAST+ suite and optimized to find long cross-species alignments of highly similar sequences [94]. An “ortholog pair” was declared if a Reference ORF and a Trinity Transcript were mutual best hits and an “ortholog set” was declared if such ortholog pairs could be identified in all species. We further refined the sequences in each ortholog set as follows: they must have a start and stop codons in at least 80% of the sequences in the set; and they must be within ±50% of the median length of the sequences in the set.

### Analysis of differential gene expression

For each species, trimmed RNA-seq reads (in FASTQ format) for each biological replicate were aligned to ortholog sets using TopHat2 [95] and read counting was performed using featureCounts [96]. Raw counts were normalized by Trimmed Mean of M-values (TMM) correction [97] using the R package edgeR [98]. Library size normalized read counts were next subjected to the voom function (variance modeling at the observation-level) function in limma R package v3.18.13 [99] with trend=TRUE for the eBayes function and correction for multiple testing (Benjamini–Hochberg false discovery rate of 0.05). Following limma analysis, strict parameters were set to denote genes as differentially expressed between the bowhead whale and other mammals: significant genes required a log2 fold-change of at least 2.0, a Benjamini Hochberg-adjusted P-value less than or equal to 0.05, and a B-value of least 2.945; representing a 95% probability that each gene was differentially expressed.

### Phylogenetic analysis

Orthologs identified from ten *de novo* liver transcriptome assemblies were joined into one ‘super gene’ for each species. Briefly, nucleotide sequences were aligned with ClustalO v1.2.1 [100] and trimmed using Gblocks v0.91b, preserving codon information [101, 102]. A ML (Maximal Likelihood) phylogenetic tree was constructed in RAxML8.0.0 [103] using all codons included and the first and second codon respectively. The best likelihood trees were searched under the GTR-GAMMA model with six categories of rate variation (500 bootstrap replicates were undertaken for estimation of node support). Bayesian molecular dating was adopted to estimate species divergence time using MCMCTree implemented in PAML (v4.4b) [104]. For each Bayesian analysis, 2,000,000 generations of MCMC (Markov chain Monte Carlo) analysis of phylogenetic trees were performed, with the first 2,000 generations discarded as burn-in. The remaining trees were sampled every 100 generations to build consensus trees. Calibration times (50-60 Mya) of divergence between Cetacea (includes the bowhead whale and minke whale) and Artiodactyla (includes the cow and yak) were obtained from fossil records [2, 3].

### Identification of proteins with unique amino acid changes

To obtain single best orthologs to human RefSeq proteins, as in the UCSC human 100 species multiple alignment track [105], Trinity FASTA files of the bowhead whale kidney (n=4) and liver (n=3) were concatenated and putative open reading frames (ORFs) identified using the Perl script TransDecoder bundled with Trinity [13]. Next, to associate predicted bowhead whale peptide sequences with human RefSeq IDs, tBLASTn v2.2.29+ of the BLAST+ suite [94] with an E-value cut-off set at 1e-5 was employed. The best match (>50% overall amino acid sequence identity along the entire sequence and spanning >75% of the length of the query sequence) was used to annotate the sequences. Protein sequences from the the toothed whales the bottlenose dolphin (*Tursiops truncatus*) and the killer whale (*Orcinus orca*) were available in the UCSC multiple alignment track. The genomes of an additional baleen whale, the minke whale (*Balaenoptera acutorostrata*) [8] (GenBank Assembly GCF_000493695.1), and two toothed whales: the sperm whale (*Physeter catodon*) (NCBI Assembly GCF_000472045.1) and the Yangtze River dolphin (*Lipotes vexillifer*) [11] (GenBank Assembly GCF_000442215.1) were queried using human UCSC multiway coding sequences and gmap v2014-07-28 (a genomic mapping and alignment program for mRNA and EST sequences) [106] with the parameters --cross-species --align --direction=sense_force -Y. The gmap output was parsed using custom Perl scripts. Bowhead whale proteins were aligned to orthologs from 67 vertebrate species (Supplemental Table 7) using ClustalO v1.2.1 [100].

In-house Perl scripts were used to parse the ClustalO output and identify unique amino acids. A Perl script scanned orthologous proteins for sites where types/groups of residues unique to the bowhead whale. The script groups residues into four groups: acidic (ED), basic (KHR), cysteine (C) and “other” (STYNQGAVLIFPMW). For example, it identifies cases where the bowhead whale harbors E or D, whilst the other organisms contain exclusively basic, cysteine, or “other” residues at that particular site. The false positive rate of the detection of unique amino acids is approximately 1.33 per analysis; or in other words, one false positive per 315 unique amino acid residue candidates [39]. To validate the results pertaining to specific genes, we manually inspected multiple sequence alignments and interrogated raw bowhead whale transcriptome data for selected genes. We appreciate that, in lieu of lack of bowhead whale genome data, unique amino acid inferences are putative when supported by a limited number of RNA-sequencing reads.

### Identification of genes under positive selection in the bowhead whale

The orthologous gene set (9,395 protein-coding genes) identified in our R pipeline was aligned by ClustalO [100]. We next employed Gblocks [101, 102] to minimise the impact of multiple sequence alignment errors and divergent regions. We used the program CodeML in the PAML v4.6 package to perform the optimized branch-site test [104, 107], as described previously [39]. Briefly, we compared PAML modelA1 (where codons evolve neutrally and under purifying selection) with ModelA (where codons on the branch of interest can be under positive selection). Following PAML analysis, likelihood ratio test (LRT) P-values were computed assuming that the null distribution was a chi-squared distribution with 1 degree of freedom at a false discovery rate of 0.05. We applied a sequential Bonferroni correction to account for the multiple comparisons made in these analyses. Manual inspection of positive selection data is currently recommended in the literature [108, 109], and we manually examined all significant alignments.

## SUPPLEMENTAL DATA TABLES


