# Supplementary material for: The transcriptome of the bowhead whale Balaena mysticetus reveals adaptations of the longest-lived mammal
Source: Aging (Albany NY). 2014 Nov 3;6(10):879–99. doi: 10.18632/aging.100699 (PMC4247388; doi:10.18632/aging.100699)
Supplement: Supplementary file 1 [file aging-06-879-s001.pdf]

SUPPLEMENTAL DATA

Supplemental Table 1. Summary of bowhead whale tissues. Animals were captured by Alaskan Iñupiaq Eskimos during the 2010 subsistence hunt in Barrow, Alaska.

Whale ID	Date	Length (m)	Sex	Sequenced tissue(s)
10B15	7 Oct 2010	12.5	F	kidney
10B16	7 Oct 2010	7.9	M	kidney, liver, heart
10B18	8 Oct 2010	9.1	F	kidney, liver
10B21	9 Oct 2010	11.5	M	kidney, liver

Supplemental Table 2. Trinity *de novo* assembly statistics. N50 is the shortest sequence (in bp) in a set of sequences that together cover 50% of the total length of all assembled sequences.

**KIDNEY**			
**Species**	**Total assembled bases**	**Average contig length**	**Contig N50**
bowhead whale	341,207,982	827.0	1,757
Brandt's bat	111,480,415	659.1	1,184
cow	222,232,281	687.1	1,283
minke whale	125,846,350	830.5	1,845
mouse	202,736,316	551.7	711
naked mole rat	115,089,648	602.5	883
rat	198,547,815	582.2	838
rhesus	244,323,096	548.3	688
Chinese tree shrew	73,017,424	677.6	1,186
**LIVER**			
**Species**	**Total assembled bases**	**Average contig length**	**Contig N50**
bowhead whale	267,997,432	851.2	1,836
Brandt's bat	75,390,009	623.2	978

cow	195,515,095	706.8	1,290
minke whale	96,531,396	727.1	1,376
mouse	166,651,410	929.0	929
naked mole rat	109,558,846	579.3	802
rat	163,138,099	637.8	1,009
rhesus	225,165,355	563.6	763
Chinese tree shrew	51,457,297	728.1	1,234
yak	61,713,325	608.5	859
**HEART**			
**Species**	**Total assembled bases**	**Average contig length**	**Contig N50**
bowhead whale	49,542,996	635.4	1,006
cow	71,474,262	846.4	1,776
minke whale	89,129,137	855.8	1,863
mouse	14,454,056	528.0	654
rat	123,905,647	603.7	962
rhesus	15,679,757	517.3	623
Chinese tree shrew	41,680,276	672.7	1,000
yak	73,020,901	610.3	866

Supplemental Table 3. Genes differentially expressed in the Brandt's bat liver compared to other mammals.

For each gene, gene counts were normalized across all replicates. We used an absolute value of log2 Ratio ≥ 2, a Benjamini-Hochberg corrected P-value ≤ 0.05, and a B-value of at least 2.945 (representing a 95% probability that a gene is differentially expressed) as the threshold to judge the significance of gene expression difference between the Brandt's bat and other mammals. A negative fold change denotes a higher gene expression compared to the other mammals examined, and vice versa.

Gene symbol (mouse)	Ensembl transcript ID	Name	logFC	P-value	BH-adj. P-value	B-value
0610037 L13Rik	ENSMUST00000125107	RIKEN cDNA 0610037L13 gene	3.56	1.99E-06	4.31E-04	5.05
Adh1	ENSMUST00000004232	alcohol dehydrogenase 1 (class I)	6.81	1.38E-10	7.51E-07	13.85
Aff1	ENSMUST00000031256	AF4/FMR2 family, member 1	-3.37	3.19E-06	5.03E-04	4.61
Agpat6	ENSMUST00000167004	1-acylglycerol-3-phosphate O-acyltransferase 6 (lysophosphatidic acid acyltransferase, zeta)	4.80	4.17E-06	5.61E-04	4.35
Aldh2	ENSMUST00000031411	aldehyde dehydrogenase 2, mitochondrial	3.06	2.60E-07	1.05E-04	7.00
Arhgef3	ENSMUST00000049206	Rho guanine nucleotide exchange factor (GEF) 3	-4.90	1.98E-07	8.59E-05	7.25
Ate1	ENSMUST00000033139	arginyltransferase 1	-2.35	1.12E-05	1.01E-03	3.41
Bin1	ENSMUST00000025239	bridging integrator 1	3.23	1.28E-06	3.51E-04	5.46
Cbr4	ENSMUST00000034058	carbonyl reductase 4	3.49	1.76E-05	1.36E-03	3.00
Cdc42bp a	ENSMUST00000111117	CDC42 binding protein kinase alpha	-4.07	1.37E-08	1.12E-05	9.76
Cog7	ENSMUST00000057576	component of oligomeric golgi complex 7	-2.16	1.28E-05	1.08E-03	3.28
Cpn1	ENSMUST00000026210	carboxypeptidase N, polypeptide 1	-3.82	1.24E-05	1.06E-03	3.31
Cpne3	ENSMUST00000029885	copine III	-2.25	8.17E-06	8.39E-04	3.71
Creb1	ENSMUST00000049932	cAMP responsive element binding protein 1	-2.38	2.68E-06	4.87E-04	4.77
Dcaf13	ENSMUST00000022909	DDB1 and CUL4 associated factor 13	-3.79	4.81E-09	6.79E-06	10.74
Dym	ENSMUST00000039608	dymeclin	-2.60	1.81E-06	4.25E-04	5.15
Erbb3	ENSMUST00000082059	v-erb-b2 erythroblastic leukemia viral oncogene homolog 3	4.62	6.32E-08	3.96E-05	8.25

		(avian)				
Etnk2	ENSMUST00000135222	ethanolamine kinase 2	-3.45	2.67E-06	4.87E-04	4.77
Ewsr1	ENSMUST00000102930	Ewing sarcoma breakpoint region 1	6.58	2.66E-10	7.51E-07	13.20
Fam195a	ENSMUST00000026828	family with sequence similarity 195, member A	-3.27	1.92E-06	4.31E-04	5.09
Fam20b	ENSMUST00000122424	family with sequence similarity 20, member B	-2.73	5.53E-06	6.51E-04	4.08
Fars2	ENSMUST00000021857	phenylalanine-tRNA synthetase 2 (mitochondrial)	3.34	4.90E-06	6.29E-04	4.20
Farsa	ENSMUST00000003906	phenylalanyl-tRNA synthetase, alpha subunit	-2.16	6.02E-06	6.78E-04	4.00
Foxk2	ENSMUST00000106113	forkhead box K2	2.91	6.77E-06	7.21E-04	3.90
Foxo1	ENSMUST00000053764	forkhead box O1	-2.75	1.11E-05	1.01E-03	3.42
Hadhb	ENSMUST00000026841	hydroxyacyl-Coenzyme A dehydrogenase/3-ketoacyl-Coenzyme A thiolase/enoyl-Coenzyme A hydratase (trifunctional protein), beta subunit	2.30	1.44E-05	1.16E-03	3.17
Hao1	ENSMUST00000028704	hydroxyacid oxidase 1, liver	3.88	1.30E-06	3.51E-04	5.45
Hif1an	ENSMUST00000040455	hypoxia-inducible factor 1, alpha subunit inhibitor	-2.85	3.14E-06	5.03E-04	4.62
Hmgn1	ENSMUST00000050884	high mobility group nucleosomal binding domain 1	3.11	1.66E-05	1.31E-03	3.05
Hnrnpa2b1	ENSMUST00000114459	heterogeneous nuclear ribonucleoprotein A2/B1	2.27	8.14E-06	8.39E-04	3.71
Irf6	ENSMUST00000076521	interferon regulatory factor 6	2.49	3.84E-06	5.29E-04	4.42
Ldlr	ENSMUST00000034713	low density lipoprotein receptor	-3.46	1.75E-06	4.25E-04	5.18
Lipa	ENSMUST00000049572	lysosomal acid lipase A	4.30	3.14E-07	1.18E-04	6.77
Man2c1	ENSMUST00000160147	mannosidase, alpha, class 2C, member 1	5.10	7.62E-09	8.60E-06	10.19
Maob	ENSMUST00000040820	monoamine oxidase B	3.70	8.80E-08	4.97E-05	7.98
Mapk1ip1l	ENSMUST00000166743	mitogen-activated protein kinase 1 interacting protein 1-like	-3.18	5.71E-06	6.58E-04	4.05
Mfsd7c	ENSMUST00000040461	major facilitator superfamily domain containing 7C	-3.95	1.32E-05	1.09E-03	3.25
Mon1a	ENSMUST00000035202	MON1 homolog A (yeast)	3.65	9.75E-06	9.59E-04	3.55
Mxd4	ENSMUST00000042701	Max dimerization protein 4	3.55	1.31E-06	3.51E-04	5.44
Myl6	ENSMUST00000164181	myosin, light	2.69	2.30E-06	4.81E-04	4.92

		polypeptide 6, alkali, smooth muscle and non-muscle				
Nap1l1	ENSMUST00000171797	nucleosome assembly protein 1-like 1	3.70	2.90E-06	5.03E-04	4.69
Osgin1	ENSMUST00000152420	oxidative stress induced growth inhibitor 1	4.25	9.21E-06	9.29E-04	3.61
Paip2	ENSMUST00000041314	polyadenylate-binding protein-interacting protein 2	2.62	1.68E-05	1.31E-03	3.05
Parva	ENSMUST00000106643	parvin, alpha	4.23	1.39E-08	1.12E-05	9.64
Pdzd8	ENSMUST00000099274	PDZ domain containing 8	-3.13	5.32E-06	6.48E-04	4.12
Pgp	ENSMUST00000053024	phosphoglycolate phosphatase	-3.15	1.22E-05	1.06E-03	3.33
Pigg	ENSMUST00000119014	phosphatidylinositol glycan anchor biosynthesis, class G	-2.60	6.53E-06	7.09E-04	3.92
Pld3	ENSMUST00000117095	phospholipase D family, member 3	-4.67	1.55E-07	7.31E-05	7.48
Pnpla2	ENSMUST00000164016	patatin-like phospholipase domain containing 2	-3.54	3.46E-06	5.03E-04	4.53
Pofut1	ENSMUST00000049863	protein O-fucosyltransferase 1	-2.85	5.39E-06	6.48E-04	4.11
Pptc7	ENSMUST00000053426	PTC7 protein phosphatase homolog (S. cerevisiae)	-3.35	2.50E-06	4.86E-04	4.84
Rbm48	ENSMUST00000042753	RNA binding motif protein 48	2.24	1.08E-05	1.00E-03	3.46
Rbp4	ENSMUST00000025951	retinol binding protein 4, plasma	4.77	8.04E-10	1.51E-06	12.41
Reep4	ENSMUST00000047218	receptor accessory protein 4	-4.78	9.92E-08	5.09E-05	7.90
Rgn	ENSMUST00000023832	regucalcin	5.23	1.33E-05	1.09E-03	3.26
Rnf8	ENSMUST00000024817	ring finger protein 8	3.51	2.43E-06	4.86E-04	4.86
Sat1	ENSMUST00000112551	spermidine/spermine N1-acetyl transferase 1	4.74	3.48E-06	5.03E-04	4.52
Secisbp2	ENSMUST00000040117	SECIS binding protein 2	-2.53	9.75E-07	3.06E-04	5.74
Sgk2	ENSMUST00000018012	serum/glucocorticoid regulated kinase 2	-4.02	6.89E-07	2.29E-04	6.07
Slc43a1	ENSMUST00000028469	solute carrier family 43, member 1	-3.76	5.11E-06	6.41E-04	4.16
Slc9a1	ENSMUST00000030669	solute carrier family 9 (sodium/hydrogen exchanger), member 1	-3.19	1.42E-06	3.63E-04	5.38
Slco1b2	ENSMUST00000042812	solute carrier organic anion transporter family, member 1b2	4.96	1.91E-08	1.35E-05	9.44
Snrnp27	ENSMUST00000113683	small nuclear ribonucleoprotein 27 (U4/U6.U5)	2.22	1.87E-05	1.41E-03	2.94
Steap3	ENSMUST00000112643	STEAP family member 3	-3.01	1.23E-05	1.06E-03	3.32
Sugt1	ENSMUST00000054908	SGT1, suppressor of G2 allele of SKP1 (S.	2.38	1.06E-05	1.00E-03	3.47

		cerevisiae)				
Tat	ENSMUST00000001720	tyrosine aminotransferase	-3.60	3.33E-06	5.03E-04	4.56
Terf1	ENSMUST00000188371	telomeric repeat binding factor 1	2.97	6.84E-07	2.29E-04	6.04
Thap1	ENSMUST00000036807	THAP domain containing, apoptosis associated protein 1	-2.41	6.13E-06	6.78E-04	3.99
Tmem131	ENSMUST00000027290	transmembrane protein 131	-4.20	9.86E-06	9.59E-04	3.53
Tpra1	ENSMUST00000055022	transmembrane protein, adipocyte asscociated 1	-2.75	4.72E-06	6.20E-04	4.23
Wipf2	ENSMUST00000037480	WAS/WASL interacting protein family, member 2	-3.22	1.01E-05	9.63E-04	3.52
Wnk1	ENSMUST00000088644	WNK lysine deficient protein kinase 1	-3.06	3.20E-06	5.03E-04	4.60
Zak	ENSMUST00000090824	sterile alpha motif and leucine zipper containing kinase AZK	-2.80	3.73E-06	5.26E-04	4.46
Zdhhc17	ENSMUST00000041723	zinc finger, DHHC domain containing 17	2.34	3.22E-06	5.03E-04	4.59

Supplemental Table 4. Enrichment of biological process (BP) Gene Ontology (GO) terms for genes differentially expressed in the Brandt's bat liver compared to other mammals.

GO Category	Term	Count	Genes	Fold Enrichment	Fisher's exact P-value
GO:0006432	phenylalanyl-tRNA aminoacylation	2	*Fars2, Farsa*	115.2	1.10E-04
GO:0046486	glycerolipid metabolic process	4	*Agpat6, Pigg, Pnpla2, Etnk2*	7.1	2.40E-03
GO:0016042	lipid catabolic process	4	*Pld3, Lipa, Pnpla2, Hadhb*	6.9	2.70E-03
GO:0045017	glycerolipid biosynthetic process	3	*Aagat6, Pigg, Etnk2*	11.9	2.00E-03
GO:0009725	response to hormone stimulus	4	*Rbp4, Adh1, Foxo1, Tat*	5.6	5.70E-03
GO:0042157	lipoprotein metabolic process	3	*Zdhhc17, Ldlr, Pigg*	9.1	4.40E-03
GO:0042572	retinol metabolic process	2	*Rbp4, Adh1*	46.1	8.20E-04
GO:0009719	response to endogenous stimulus	4	*Rbp4, Adh1, Foxo1, Tat*	5.0	8.30E-03
GO:0008654	phospholipid biosynthetic process	3	*Agpat6, Pigg, Etnk2*	8.3	5.60E-03
GO:0030879	mammary gland development	3	*Agpat6, Irf6, Creb1*	8.0	6.20E-03
GO:0010033	response to organic substance	6	*Rbp4, Adh1, Creb1, Foxo1, Tat, Slc9a1*	2.7	2.20E-02
GO:0055114	oxidation reduction	7	*Hao1, Steap3, Hif1an, Adh1, Maob, Aldh2, Cbr4*	2.4	2.60E-02
GO:0008283	cell proliferation	4	*Rbp4, Lipa, Irf6, Tthap1*	3.7	2.20E-02
GO:0009306	protein secretion	2	*Steap3, Mon1a*	20.9	4.00E-03
GO:0009628	response to abiotic stimulus	4	*Hmgn1, Rnf8, Rbp4, Slc9a1*	3.7	2.30E-02

Supplemental Table 5. Genes differentially expressed in the naked mole rat liver compared to other mammals.

For each gene, gene counts were normalized across all replicates. We used an absolute value of log2 Ratio ≥ 2, a Benjamini-Hochberg corrected P-value ≤ 0.05, and a B-value of at least 2.945 (representing a 95% probability that a gene is differentially expressed) as the threshold to judge the significance of gene expression difference between the naked mole rat and other mammals. A negative fold change denotes a higher gene expression compared to the other mammals examined, and vice versa.

Gene symbol (mouse)	Ensembl transcript ID	Name	logFC	P-value	BH-adj. P-value	B-value
Acap2	ENSMUST00000058033	ArfGAP with coiled-coil, ankyrin repeat and PH domains 2	-2.20	1.27E-05	1.56E-03	3.30
Afm	ENSMUST00000113179	afamin	5.43	1.50E-06	4.47E-04	5.29
Angptl3	ENSMUST00000030280	angiopoietin-like 3	-2.97	4.22E-06	8.99E-04	4.35
Arhgap32	ENSMUST00000174641	Rho GTPase activating protein 32	-4.30	1.58E-05	1.78E-03	3.09
Arl3	ENSMUST00000026009	ADP-ribosylation factor-like 3	-2.47	5.86E-07	2.54E-04	6.22
Cask	ENSMUST00000115438	calcium/calmodulin-dependent serine protein kinase (MAGUK family)	-2.93	9.89E-06	1.35E-03	3.54
Ccdc12	ENSMUST00000019803	coiled-coil domain containing 12	2.73	5.19E-07	2.44E-04	6.29
Cct6a	ENSMUST00000031402	chaperonin containing Tcp1, subunit 6a (zeta)	5.86	5.54E-13	3.13E-09	18.33
Cops5	ENSMUST00000027050	COP9 (constitutive photomorphogenic) homolog, subunit 5 (Arabidopsis thaliana)	2.58	4.30E-06	8.99E-04	4.32
Cox15	ENSMUST00000045562	cytochrome c oxidase assembly protein 15	2.42	6.48E-06	1.08E-03	3.94
Crtc3	ENSMUST00000122255	CREB regulated transcription coactivator 3	-3.14	9.01E-06	1.35E-03	3.63
D930015E06Rik	ENSMUST00000052342	RIKEN cDNA D930015E06 gene	4.56	1.24E-06	4.15E-04	5.49
Deaf1	ENSMUST00000080553	deformed epidermal autoregulatory factor 1 (Drosophila)	-3.29	2.08E-07	1.21E-04	7.19
Dmtf1	ENSMUST00000071921	cyclin D binding myb-like transcription factor 1	-2.29	9.76E-06	1.35E-03	3.55
Dtwd1	ENSMUST00000170908	DTW domain containing 1	-3.15	5.73E-06	1.07E-03	4.06

Fam168b	ENSMUST00000167518	family with sequence similarity 168, member B	-3.26	2.15E-07	1.21E-04	7.17
Fto	ENSMUST00000069718	fat mass and obesity associated	3.35	1.01E-05	1.35E-03	3.53
Gabpb1	ENSMUST00000110425	GA repeat binding protein, beta 1	-2.62	3.24E-08	3.66E-05	8.92
Hgsnat	ENSMUST00000037609	heparan-alpha-glucosaminide N-acetyltransferase	3.18	1.25E-06	4.15E-04	5.47
Hpcal1	ENSMUST00000071858	hippocalcin-like 1	-3.41	1.68E-07	1.21E-04	7.40
Llph	ENSMUST00000020444	LLP homolog, long-term synaptic facilitation (Aplysia)	-3.46	3.23E-08	3.66E-05	8.94
Mef2d	ENSMUST00000107559	myocyte enhancer factor 2D	3.21	1.12E-06	4.15E-04	5.58
Mex3c	ENSMUST00000091852	mex3 homolog C (C. elegans)	3.13	2.56E-06	6.57E-04	4.81
Minos1	ENSMUST00000143971	mitochondrial inner membrane organizing system 1	2.46	1.78E-05	1.89E-03	2.99
N6amt2	ENSMUST00000022518	N-6 adenine-specific DNA methyltransferase 2 (putative)	-3.60	4.89E-06	9.87E-04	4.21
Pced1a	ENSMUST00000089581	PC-esterase domain containing 1A	3.87	1.37E-06	4.30E-04	5.39
Pin4	ENSMUST00000113627	protein (peptidyl-prolyl cis/trans isomerase) NIMA-interacting, 4 (parvulin)	-2.01	1.00E-05	1.35E-03	3.52
Pms1	ENSMUST00000027267	postmeiotic segregation increased 1 (S. cerevisiae)	-3.22	8.54E-06	1.35E-03	3.68
Polr3f	ENSMUST00000028914	polymerase (RNA) III (DNA directed) polypeptide F	-2.61	1.60E-06	4.52E-04	5.25
Prkab1	ENSMUST00000031486	protein kinase, AMP-activated, beta 1 non-catalytic subunit	-2.65	1.51E-05	1.76E-03	3.13
Proc	ENSMUST00000171765	protein C	2.77	4.74E-07	2.43E-04	6.42
Psmb8	ENSMUST00000025196	proteasome (prosome, macropain) subunit, beta type 8 (large multifunctional peptidase 7)	4.51	7.28E-09	2.05E-05	10.21
Rad17	ENSMUST00000022136	RAD17 homolog (S. pombe)	-2.67	2.21E-06	5.93E-04	4.96
Rnf144b	ENSMUST00000068891	ring finger protein 144B	-2.13	1.44E-05	1.73E-03	3.19
Rnf2	ENSMUST00000187048	ring finger protein 2	-2.75	6.27E-06	1.07E-03	3.97
Rpf2	ENSMUST00000183309	ribosome production factor 2 homolog (S. cerevisiae)	-2.90	4.40E-08	4.14E-05	8.65
Rufy1	ENSMUST00000020643	RUN and FYVE domain containing 1	-3.24	1.09E-08	2.05E-05	9.95
Secisbp2l	ENSMUST00000053699	SECIS binding protein 2-like	-3.48	1.23E-06	4.15E-04	5.52

Setd3	ENSMUST00000071095	SET domain containing 3	3.71	1.03E-05	1.36E-03	3.50
Slc25a30	ENSMUST00000022580	solute carrier family 25, member 30	3.14	8.99E-06	1.35E-03	3.63
Smc3	ENSMUST00000025930	structural maintenance of chromosomes 3	-2.64	1.09E-05	1.40E-03	3.44
Sox13	ENSMUST00000153799	SRY (sex determining region Y)-box 13	-3.25	4.09E-06	8.99E-04	4.38
Spice1	ENSMUST00000050897	spindle and centriole associated protein 1	-3.31	1.77E-05	1.89E-03	2.98
Ss18	ENSMUST00000040924	synovial sarcoma translocation, Chromosome 18	-2.82	1.53E-05	1.76E-03	3.12
Ssr3	ENSMUST00000029414	signal sequence receptor, gamma	2.87	1.87E-05	1.89E-03	2.95
Stx4a	ENSMUST00000033075	syntaxin 4A (placental)	-2.13	4.24E-06	8.99E-04	4.34
Tk2	ENSMUST00000050211	thymidine kinase 2, mitochondrial	-3.00	6.04E-06	1.07E-03	4.01
Uox	ENSMUST00000029837	urate oxidase	7.81	6.07E-06	1.07E-03	4.00
Vamp4	ENSMUST00000132158	vesicle-associated membrane protein 4	-3.18	2.92E-06	7.17E-04	4.69
Zfp292	ENSMUST00000047950	zinc finger protein 292	-6.12	1.74E-07	1.21E-04	7.37

Supplemental Table 6. Enrichment of biological process (BP) Gene Ontology (GO) terms for genes differentially expressed in the naked mole rat liver compared to other mammals.

GO Category	Term	Count	Genes	Fold Enrichment	Fisher's exact P-value
GO:0007049	cell cycle	5	*DMTF1, RNF2, SMC3, RAD17, ARL3*	3.3	1.70E-02
GO:0006259	DNA metabolic process	4	*TK2, SMC3, RAD17, PMS1*	3.8	2.00E-02

Supplemental Table 7. Overview of species examined in the unique amino acid change analysis.

Common name	Binomial name
aardvark	*Orycteropus afer afer*
African elephant	*Loxodonta africana*
alpaca	*Vicugna pacos*
armadillo	*Dasypus novemcinctus*
baboon	*Papio hamadryas*
Bactrian camel	*Camelus ferus*
big brown bat	*Eptesicus fuscus*
black flying-fox	*Pteropus alecto*
bottlenose dolphin	*Tursiops truncates*
bowhead whale	*Balaena mysticetus*
brush-tailed rat	*Octodon degus*
bushbaby	*Otolemur garnettii*
cape elephant shrew	*Elephantulus edwardii*
cape golden mole	*Chrysochloris asiatica*
cat	*Felis catus*
chicken	Gallus gallus
chimpanzee	*Pan troglodytes*
chinchilla	*Chinchilla lanigera*
Chinese hamster	*Cricetulus griseus*
Chinese tree shrew	*Tupaia chinensis*
(common) shrew	*Sorex araneus*
cow	*Bos taurus*
crab-eating macaque	*Macaca fascicularis*
David's Myotis bat	*Myotis davidii*
dog	*Canis lupus familiaris*
European hedgehog	*Erinaceus europaeus*
ferret	*Mustela putorius furo*

gibbon	*Nomascus leucogenys*
goat	*Capra hircus*
golden hamster	*Mesocricetus auratus*
gorilla	*Gorilla gorilla gorilla*
greater flying fox	*Pteropus vampyrus*
green monkey	*Chlorocebus sabaeus*
guinea pig	*Cavia porcellus*
horse	*Equus caballus*
human	*Homo sapiens*
killer whale	*Orcinus orca*
lesser Egyptian jerboa	*Jaculus jaculus*
little brown bat	*Myotis lucifugus*
manatee	*Trichechus manatus latirostris*
marmoset	*Callithrix jacchus*
minke whale	*Balaenoptera acutorostrata*
mouse	*Mus musculus*
naked mole rat	*Heterocephalus glaber*
opossum	*Monodelphis domestica*
orangutan	*Pongo pygmaeus abelii*
Pacific walrus	*Odobenus rosmarus divergens*
panda	*Ailuropoda melanoleuca*
pig	*Sus scrofa*
pika	*Ochotona princeps*
platypus	*Ornithorhynchus anatinus*
prairie vole	*Microtus ochrogaster*
rabbit	*Oryctolagus cuniculus*
rat	*Rattus norvegicus*
rhesus macaque	*Macaca mulatta*
sheep	*Ovis aries*
sperm whale	*Physeter catodon*
squirrel monkey	*Saimiri boliviensis*
star-nosed mole	*Condylura cristata*
Tasmanian devil	*Sarcophilus harrisii*
tenrec	*Echinops telfairi*
thirteen-lined ground squirrel	*Spermophilus tridecemlineatus*
Tibetan antelope	*Pantholops hodgsonii*
wallaby	*Macropus eugenii*
Weddell seal	*Leptonychotes weddellii*
Western clawed frog	*Xenopus tropicalis*
white rhinoceros	*Ceratotherium simum*
Yangtze River dolphin	*Lipotes vexillifer*
